# Clustering with position-specific constraints on variance: Applying redescending M-estimators to label-free LC-MS data analysis

**DOI:** 10.1186/1471-2105-12-358

**Published:** 2011-08-31

**Authors:** Rudolf Frühwirth, D R Mani, Saumyadipta Pyne

**Affiliations:** 1 Institute of High Energy Physics, Austrian Academy of Sciences, Vienna, Austria; 2 Broad Institute of MIT and Harvard University, Cambridge, MA, USA; 3 Dana-Farber Cancer Institute, Harvard Medical School, Boston, MA, USA

## Abstract

**Background:**

Clustering is a widely applicable pattern recognition method for discovering groups of similar observations in data. While there are a large variety of clustering algorithms, very few of these can enforce constraints on the variation of attributes for data points included in a given cluster. In particular, a clustering algorithm that can limit variation within a cluster according to that cluster's position (centroid location) can produce effective and optimal results in many important applications ranging from clustering of silicon pixels or calorimeter cells in high-energy physics to label-free liquid chromatography based mass spectrometry (LC-MS) data analysis in proteomics and metabolomics.

**Results:**

We present MEDEA (M-Estimator with DEterministic Annealing), an M-estimator based, new unsupervised algorithm that is designed to enforce position-specific constraints on variance during the clustering process. The utility of MEDEA is demonstrated by applying it to the problem of "peak matching"--identifying the common LC-MS peaks across multiple samples--in proteomic biomarker discovery. Using real-life datasets, we show that MEDEA not only outperforms current state-of-the-art model-based clustering methods, but also results in an implementation that is significantly more efficient, and hence applicable to much larger LC-MS data sets.

**Conclusions:**

MEDEA is an effective and efficient solution to the problem of peak matching in label-free LC-MS data. The program implementing the MEDEA algorithm, including datasets, clustering results, and supplementary information is available from the author website at http://www.hephy.at/user/fru/medea/.

## Background

Protein or peptide biomarkers offer great promise in early detection, monitoring and targeted treatment of diseases. Two main strategies have been employed in proteomic biomarker discovery, identity-based and pattern-based methods. Identity-based methods use high quality tandem mass spectrometry (LC-MS/MS) and identify potential biomarkers among the sequenced peptides [1-3]. While identity makes the task of biomarker validation easier, the approach ignores unidentified peaks in the mass spectra resulting in significant information loss, and has limited throughput due to the need for extensive fractionation. Pattern-based, or label-free approaches [4-6], on the other hand, look for discriminating peak patterns in mass spectra, without regard to their identity. While initial attempts at pattern-based biomarker discovery using low quality instrumentation and improper validation were met with criticism [7,8], the approach nonetheless has merit [9]. Indeed the design and implementation of the PEPPeR platform for proteomic biomarker discovery [10] was an attempt to distill the best of both worlds in a robust, high throughput analytical platform for biomarker discovery. It combined both identity and pattern based approaches to capitalize on the merits of each, while exploiting synergies to minimize the drawbacks, enhancing our ability to successfully find and validate biomarkers.

PEPPeR uses high resolution and high mass accuracy liquid chromatography-based mass spectrometry (LC-MS) data from state-of-the-art mass spectrometers, and appropriately combines pattern-based (unidentified peptide peaks) and identity-based (peptides sequenced via MS/MS, or tandem mass spectrometry) information to generate peptide quantitation for biomarker discovery. From a computational standpoint, the uniqueness of this approach stems from the use of: (i) identified peptides to set automatically calculated matching tolerances for guiding the alignment of unidentified peaks; (ii) matching unidentified peaks across multiple samples (peak matching) using mixture model based clustering. In the present study, we introduce a new algorithm MEDEA (M-Estimator with DEterministic Annealing) that can enhance the analytical capacity of the PEPPeR platform. Using two real-life LC-MS datasets, and a robust statistical approach, we show how MEDEA can provide a more accurate and efficient solution to the problem of peak matching.

### The PEPPeR algorithm

A key challenge in the design of PEPPeR is the implementation of peak matching. An LC-MS peak is identified by a mass-to-charge ratio *m*/*z*, its LC retention time RT and its charge *z *[10]. The presence of a specific peptide in a sample analyzed by LC-MS will result in a peak at a given (*m*/*z*, RT, *z*) location. The intensity of the peak reflects the peptide abundance in the sample.

Due to the inherent limits of chromatography and mass spectrometry, repeated measurements of the same peptide, or measurements of the same peptide in multiple samples will result in variations in the determined *m*/*z *and RT values. The *m*/*z *variation is dictated by the mass accuracy of the mass spectrometer. For successful application of pattern-based approaches like PEPPeR, high mass accuracy is required in order to distinguish the many peptides that arise in the analysis of real-world samples. Acceptable *m*/*z *variation for PEPPeR ranges from a few parts per million (ppm) to a few 10's of ppm [10], and is easily achieved by instruments such as the LTQ OrbiTrap [11]. It is worth noting that *m*/*z *variation is a function of the actual *m*/*z *value--a ppm precision specification allows for larger variation when the *m*/*z *values are higher. Retention time variation, on the other hand, is based on chromatography and the physico-chemical properties of peptides. Typical RT variation for a peptide peak ranges from a fraction of a minute for well-behaved peptides under high performance chromatography, to several minutes under chromatographic runs extending 90-120 minutes [10]. Unlike *m*/*z *variation, RT variation can be treated as a constant limit for the entire chromatographic run.

Allowable limits of variation (tolerances) for *m*/*z *and RT are determined based on MS/MS sequenced peptides with confident identities obtained by database searching. A process called landmark matching [10] is used to propagate identities ("landmarks") across many samples, so that a subset of confidently identified peptides are present across multiple samples. Tolerances are calculated using these common landmark peptides. The upper limit of the range of variation actually observed (over multiple samples) for *m*/*z *and RT values (after excluding outliers) is defined as the variation tolerance for *m*/*z *and RT, respectively, for all peaks (sequenced or otherwise) during the peak matching process.

**Figure 1:**
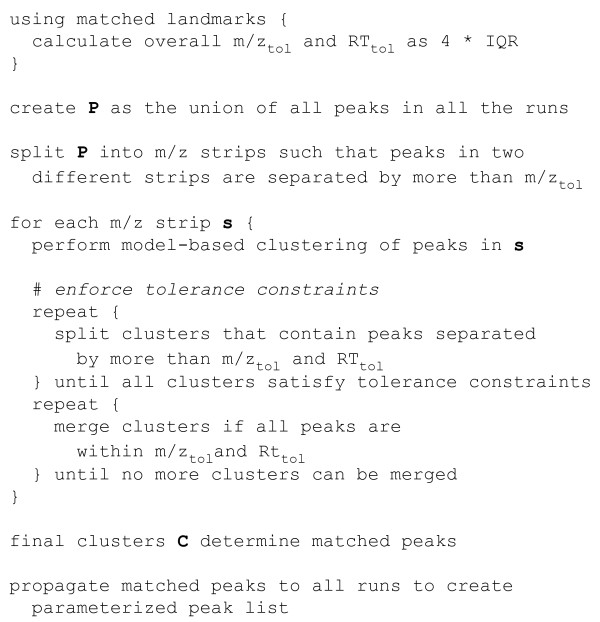
**Overview of Peak Matching**. Overview of the existing peak matching process using Gaussian mixture model-based clustering and split-merge post-processing for enforcing tolerance constraints.

Figure 1 shows an outline of the existing peak matching algorithm. Model-based clustering [12-14] implemented using a bivariate Gaussian mixture model in the MCLUST [15] library for the R statistical programming environment [16] is used to identify the "same" peak (peptide) across all the samples being analyzed. Since MCLUST is unaware of the tolerance constraints for the *m*/*z *and RT values, an iterative post-processing step is used to split and merge clusters so that the final grouping of peaks satisfies the *m*/*z *and RT tolerances. Every split and/or merge operation repeatedly invokes MCLUST. This process is independently applied to peaks with different charge states (*z*).

### The need for variance-constrained clustering

Application of MCLUST, or indeed of most current approaches, to the problem of proteomic peak matching requires the enforcement of tolerance constraints in order to limit membership to only those elements that have attributes (*m*/*z *or RT) whose values fall within the allowable variation limits (set by *m*/*z *and RT tolerances, respectively). To achieve this goal, the repeated split-merge approach used post hoc in PEPPeR can (i) result in sub-optimal clusters (see Subsection "Cluster Quality" below for an example); and can (ii) be computationally expensive and time-consuming when a large number of peaks are being clustered (see Subsection "Implementation" below).

Sub-optimal clustering can result in splitting a given peptide across multiple clusters, or conversely, merging distinct peptides into one cluster. This can result in unnecessary false positive or false negative markers, undermining the process of biomarker discovery (see Subsection "Cluster Quality"). Furthermore, split-merge based tolerance enforcement in PEPPeR has resulted in significant limitations when applying the platform to the analysis of large datasets arising from studies involving significant (several tens to a few hundred) numbers of patients. An efficient algorithm that can automatically enforce tolerance constraints during the clustering process--like the MEDEA algorithm presented here--produces more optimal clusters and enables unsupervised analysis of much larger datasets.

The MEDEA variance-constrained clustering algorithm is described in the following section. The remaining sections address the application of MEDEA to LC-MS peak matching, comparative analysis with the currently used method, followed by discussion and conclusions.

## Methods

### A redescending M-estimator with annealing

The core of the new clustering algorithm is a redescending M-Estimator with DEterministic Annealing (MEDEA). M-estimators were first introduced in [17] as robust estimators of location and scale. An M-estimator of location is obtained by minimizing a generalized objective function *ρ*(*r*):

(1) $$\tilde{\mu }=\underset{\mu }{\mathrm{argmin}}\overset{n}{\underset{i=1}{\sum }}\rho \left(\left(x_{i}-\mu \right)∕\sigma \right),$$

where *x*_1_, . . . , *x_n _*are the observations, *μ *is the location to be estimated, and *σ *is the scale of the observations, which is either known or estimated from the data. Some well-known examples of M-estimators are the *L*_2 _or least-squares estimator, with *ρ*(*r*) = *r*^2^/2; the *L*_1 _estimator, with *ρ*(*r*) = |*r*|; and Huber's M-estimator, with

(2) $$\rho \left(r\right)=\left\{\begin{array}{cc} r^{2}∕2, & |r|\leq c,\\ cr-c^{2}∕2, & |r|>c. \end{array}\right)$$

It is easy to see that an M-estimator can be computed by an iterated reweighted least-squares estimator with the following weights:

(3) $$w_{i}=\frac{\psi \left(r_{i}\right)}{r_{i}},$$

where *r_i _*= (*x_i _*- *μ*)/*σ *and *ψ*(*r*) = d*ρ*/d*r*.

A special class of M-estimators is formed by redescending M-estimators. They are widely used for robust regression and regression clustering, e.g. see [18,19] and the references therein. According to the definition in [20], the *ψ*-function of a redescending M-estimators has to disappear outside a certain central interval. In the following, we merely demand that the *ψ*-function tends to zero for |*r*| → ∞. If *ψ *tends to zero sufficiently fast, observations lying farther away than a certain bound are effectively discarded.

**Figure 2:**
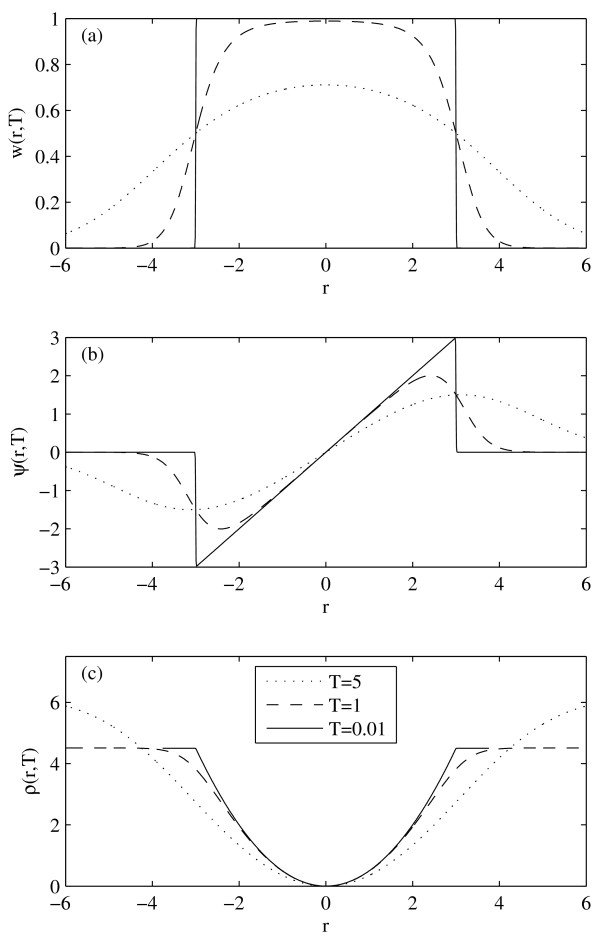
**Redescending M-estimator Characteristics**. (a) weight function; (b) *ψ*-function; (c) *ρ*-function of the redescending M-estimator in Eq. (4), for *T *= 5, 1, 0.1. The cutoff is at *c *= 3.

Redescending M-estimators are thus particularly resistant to extreme outliers, but their computation is afflicted with the problem of local minima and a resulting dependence on the starting point of the iteration. The problem of convergence to a local minimum can be solved by combining the iterative computation of the M-estimate with a global optimization technique, namely deterministic annealing. For a review of deterministic annealing and its applications to clustering, classification, regression and related problems, see [21] and the references therein. The combination of M-estimators with deterministic annealing was first proposed by Li in [22]. Li's annealing M-estimators, however, have infinite asymptotic variance at low temperature, a feature that we find undesirable in our application. Instead, we use a redescending M-estimator proposed in [23]. The estimator uses the following weights:

(4) $$\begin{array}{lll} w\left(r;c,T\right) & =\frac{\varphi \left(r∕\sqrt{T}\right)}{\varphi \left(r∕\sqrt{T}\right)+\varphi \left(c∕\sqrt{T}\right)}\; & \text{(1)}\\ & =\frac{\exp\left(-r^{2}∕2T\right)}{\exp\left(-r^{2}∕2T\right)+\exp\left(-c^{2}∕2T\right)},\; & \text{(2)}\\ & \; & \text{(3)} \end{array}$$

where *φ *is the standard normal probability density function, *T *is the temperature parameter, and *c *is the cutoff parameter. The weight function, the *ψ*-function and the *ρ*-function of this estimator are shown in Figure 2, for three different temperatures (*T *= 5, 1, 0.01). Note that the weight is always equal to 0.5 for *r *= *c*.

If the temperature increases, the weight drops more slowly as a function of *r*. In the limit of infinite temperature we have

$$\underset{T\to \infty }{\lim}w\left(r;c,T\right)=\frac{1}{2},$$

for all *c*, and the M-estimator degenerates into a least-squares estimator. If the temperature drops to zero, the weight function converges to a step function, and the M-estimator approaches the skipped mean. For more details about the estimator and its influence function, see [23].

### Clustering Algorithm

The clustering algorithm, when applied to PEPPeR peak matching, has to fulfill two basic requirements. First, all peaks in a cluster should have the same charge; this is achieved by applying the algorithm independently to each subset of peaks with the same charge *z*. Second, all members of a cluster should fit into a box whose half width is specified by the *m*/*z *and RT tolerances--identical to the split-merge post-processing constraint enforcement used with MCLUST. A cluster is forced to respect these limits by setting the scale *σ_i_*, *i *∈ {*m*/*z*, RT} of the observations to *σ_i _*= *δ_i_*/3, where *δ_i _*is the half width of the box in each coordinate, and by setting the cutoff *c *to three times the scale, i.e. to the half width *δ_i_*. The weights are computed according to Eq. (4) for each coordinate and multiplied to obtain the final weights. The algorithm can be summarized as follows:

1. Select an unused peak (the seed) as a cluster center, and find all unused peaks of the same charge in a search frame of size ± 3*δ_i_*.

2. Set the iteration number to *k *= 1.

3. Set the temperature to *T *= *T_k_*.

4. Compute the weights of all peaks in the frame relative to the current cluster center.

5. Compute the new cluster center by the weighted mean of all peaks in the frame and recompute the search frame.

6. Set *k *:= *k *+ 1 and go to 3, unless the maximum number of iterations is reached.

7. Mark all peaks in the cluster as used and go to 1.

The starting temperature is *T*_1 _= 8. This temperature is sufficiently high so that the weights are non-negligible throughout the search frame constructed in Step 1. The cluster center therefore moves toward the center-of-mass of the peaks in the search frame. A higher starting temperature would just slow down the annealing without substantially changing the final clustering. In the following steps, the temperature is lowered according to a predefined annealing schedule. We have chosen an approximately exponential schedule (see [21]): *T*_2 _= 6, *T*_3 _= 4, *T*_4 _= 3, *T*_5 _= 2, *T*_6 _= 1.5, *T*_7 _= ⋯ = *T*_11 _= 1. Several steps at *T *= 1 are performed to allow convergence of the M-estimator. The final temperature *T*_12 _= 0.25 is much lower than 1 and results in a sharp cut at the boundaries of the box.

**Figure 3:**
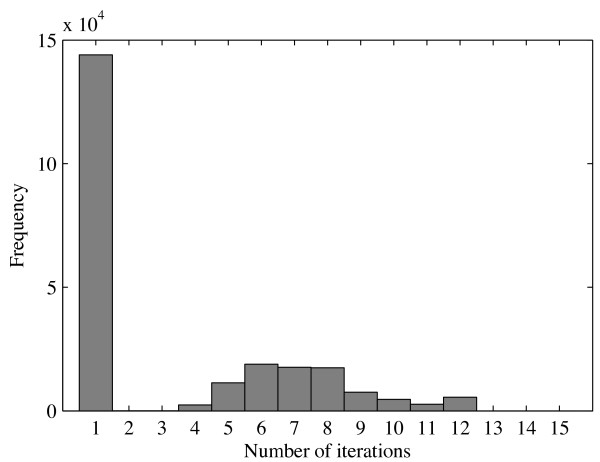
**M-estimator Annealing**. Frequency distribution of the number of annealing steps required in the computation of the M-estimator, for the DarTB dataset. Isolated clusters that respect the constraints do not require annealing and are found in a single iteration.

In many cases, the annealing can be terminated at an early stage, in order to speed up the algorithm. If at any step all peaks with weights above the threshold *w*_0 _= 0.1 are inside the box, the weights are computed immediately at the final temperature. Isolated clusters that respect the tolerances are therefore found in a single iteration. The annealing is also stopped if the cluster center does not move by more than 0.1% of the half width *δ_i _*in either coordinate. The number of iterations required for the DarTB dataset (see Subsection "Dataset Generation") is shown in the histogram in Figure 3. It can be seen that only a small fraction of clusters needs all 12 iterations.

**Figure 4:**
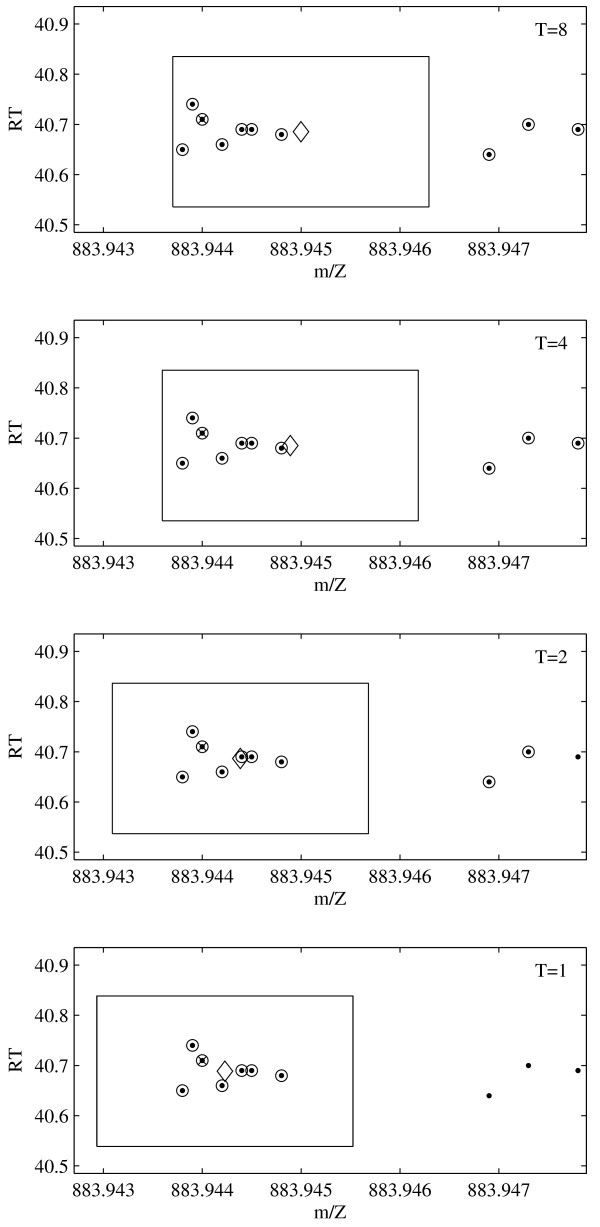
**Cluster Evolution**. Evolution of the estimated cluster center as a function of the temperature *T*. The dots are the peaks, circled dots are peaks with a weight above the threshold *w*_0 _= 0.1. The seed is marked by × , the current cluster center is marked by ◇.

At the stopping temperature only peaks inside the box have positive weights. An example of the evolution of the cluster center with falling temperature is shown in Figure 4.

It is possible to use the M-estimator without annealing, i.e., with starting temperature *T*_1 _= 1. Cohesive clusters that are well separated will still be effectively identified by the algorithm in the absence of annealing. In fact, the application to LC-MS peak clustering encounters many such isolated clusters as indicated by the large number of clusters that require only one iteration in Figure 3. But, there are a substantial number of clusters that are harder to identify, and benefit from a larger number of annealing steps. In these cases the clustering with annealing will produce different groupings, as a higher starting temperature initially explores a larger range in the data space and the cluster center is attracted more strongly to the region of highest peak density in the search frame.

### Post-processing

In the low temperature limit the M-estimator is a skipped mean, which means that cluster center is the arithmetic mean of all peaks in the box. Thus it may happen that a cluster with a few outlying points is split into two, although the entire cluster fits into a box of the prescribed size. Another shortcoming of the algorithm described above is due to its sequential nature. As the peaks attached to a cluster are not made available any more to subsequent clusters, there is no globally optimal association of peaks to clusters. In order to compensate for these shortcomings we have designed a post-processing algorithm that has two stages. In the first stage, in every region of overlapping clusters, peaks are assigned to the closest cluster center in their vicinity. In the second stage, clusters are fused if their union fits into a single box.

#### Stage 1: Globally optimal assignment

We call two clusters overlapping if their respective tolerance boxes intersect. This relation is reflexive and symmetric, but not transitive. The transitive closure of this relation is an equivalence relation *R*. Using the relation *R*, the global assignment algorithm can be described as follows:

1. Set the temperature to *T *= 1.

2. Select an unused cluster *i*.

3. Find the set *J *of all clusters *j *with (*i*, *j*) ∈ *R*.

4. Find the set *K *of all peaks in any of the clusters in *J*.

5. Compute the weights of all peaks in set *K *relative to all cluster centers in *J*.

6. Associate each peak to the cluster with the largest weight.

7. If the association has changed, recompute all cluster centers and go to 5; if not, mark all clusters in *J *as used and go to 2.

**Figure 5:**
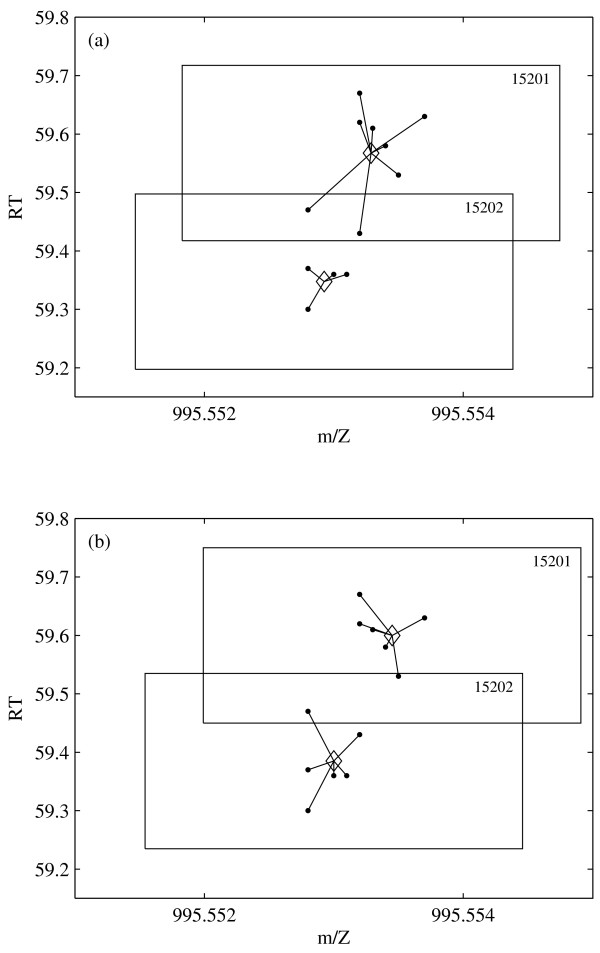
**Peak Assignment Optimization**. Example of optimizing the assignment of peaks to overlapping clusters. The dots are the peaks, the cluster centers are marked by ◇. (a) clusters before reassignment; (b) clusters after reassignment. The cluster numbers are in the right upper corner of the box.

Note that the cluster centers are recomputed not by a weighted mean, but by the mid-range in both coordinates. This guarantees that all peaks assigned to the cluster are indeed inside the box. An example with two clusters is shown in Figure 5.

#### Stage 2: Cluster fusion

The cluster fusion algorithm consists of the following steps:

1. Select an unused cluster *i*.

2. Find the set *J *of all clusters that are overlapping with *i*.

3. Compute the weights of all cluster centers in *J *relative to cluster center *i*.

4. Select the cluster center with the largest weight and call it *j*.

5. If clusters *i *and *j *fit into a single box, assign all peaks in cluster *j *to cluster *i *and drop cluster *j*.

6. Mark cluster *i *as used and go to 1.

**Figure 6:**
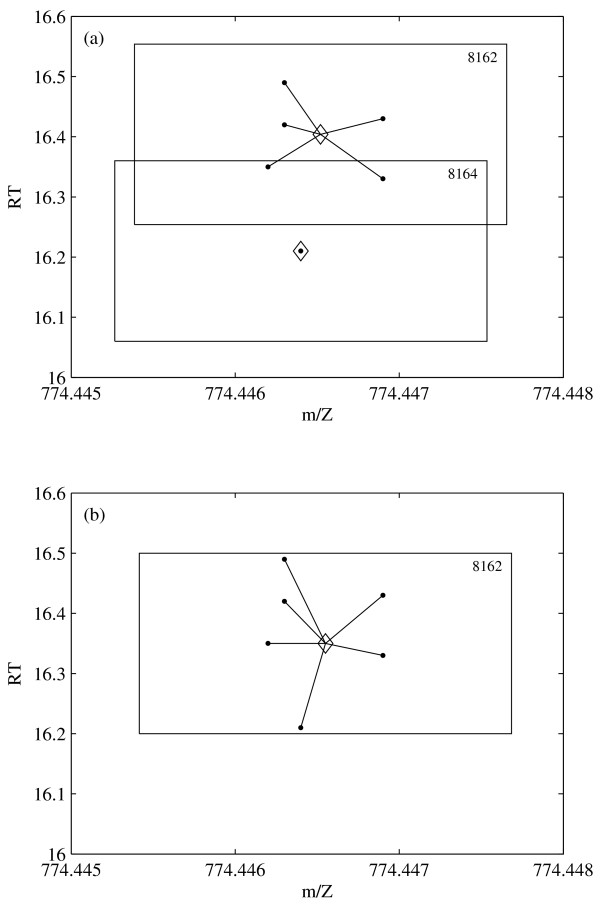
**Cluster Fusion**. Example of the fusion of two clusters. The dots are the peaks, the cluster centers are marked by ◇. (a) two clusters before fusion; (b) single cluster after fusion. The cluster numbers are in the right upper corner of the box.

If clusters *i *and *j *are fused, the new cluster center is again the mid-range of the fused cluster. An example with two clusters is shown in Figure 6.

## Results

### Dataset generation

In order to validate the effectiveness of MEDEA and to compare it with MCLUST, we have used two LC-MS datasets: (i) MitoMix: mitochondrial extracts from mice with a set of proteins spiked in at known levels [10]; and (ii) DarTB: plasma samples from tuberculosis cases and controls collected at Dar es Salaam, Tanzania, as part of the Gates Grand Challenges in Global Health GC-13 project on pattern-based proteomic characterization of the epidemiology (prevalence and incidence) of diseases of major importance in the developing world.

The MitoMix dataset was generated from mitochondrial extracts from C57BL6/J mice aged either 2 or 6 weeks, as described previously in [24]. The 2-week extract was spiked with Variability Mix *α*, the 6-week extract was spiked with Variability Mix *β *prior to digestion. The variability mixes contain 12 proteins (not found in mouse plasma) spiked in at different levels in the *α *and *β *mixes, as set out in [10]. The samples were reduced, alkylated and trypsin digested, followed by desalting and lyophilization. The samples were then reconstituted and analyzed using reverse phase LC-MS on a LTQ-FT (Thermo Scientific) mass spectrometer, with an 85 minute gradient. MS acquisition consisted of a high resolution precursor MS scan (FTMS) followed by three data-dependent MS/MS scans (ion trap) [10]. Each of the two samples (2-week extract with mix *α*, and 6 week extract with mix *β*) was run in six technical replicates to generate a total of 12 raw files that were subsequently analyzed using MCLUST and MEDEA.

The DarTB dataset consists of 20 TB case and 20 healthy control plasma samples collected at Dar es Salaam, Tanzania. The samples were shipped to The Broad Institute where they are run through a sample processing pipeline starting with immunoaffinity depletion of the top 14 abundant human proteins using an Agilent MARS-14 depletion column. The depleted plasma is passed through a low molecular weight filter and subjected to reduction, alkylation and trypsin digestion. The digested sample is then fractionated into ten fractions using a basic pH reverse phase column. Fractions 5, 6, and 7 are analyzed via LC-MS on a Thermo LTQ-FT using a 98 min gradient. The resulting 120 raw files are analyzed using MCLUST and MEDEA.

### Data processing

Raw files generated for the MitoMix and DarTB samples by LC-MS were extracted and interpreted using SpectrumMill (Agilent Technologies, CA) to provide sequence identities for peptides subject to MS/MS. The raw files were also converted to mzXML and processed using msInspect [25] for peak detection, isotope deconvolution and charge state assignment. The peaks identified by msInspect are parameterized by mass-to-charge ratio, retention time and charge (*m*/*z*, RT, *z*). These peaks are then merged with confident peptide sequence identities extracted by SpectrumMill. The result is a table of peaks--some if which are sequence identified--for each LC-MS run. The landmark matching algorithm [10] is used to propagate confident identities across samples to maximize identified peptides (landmarks) in each sample. These peaks are then subject to *m*/*z *and RT correction [10] to minimize run-to-run variation. The *m*/*z *and RT variation of common landmarks across samples are used to define *m*/*z *tolerance and RT tolerance as described in Figure 1. The peak lists from all samples in a dataset are then concatenated to generate the data table that is used for MCLUST and MEDEA analysis.

### Cluster quality

#### The MitoMix data set

The MitoMix dataset consists of a total of 92,706 peaks from all the samples and replicates. For each peak, *m*/*z*, RT and *z *are given. Based on the observed variation for known landmark peptides, the half width of the tolerance box was set to *δ*_1 _= 2.93E-6 · *m*/*z *in *m*/*z *(2.93 ppm) and *δ*_2 _= 0.3 in RT. The general characteristics of the two clustering algorithms are summarized in Table 1.

**Table 1:** MCLUST vs. MEDEA Comparison for MitoMix Data

	MCLUST	MEDEA
Number of clusters	23448	20765
Average cluster size	3.95	4.47
Average cluster diameter in *m*/*z*	6.25E-4	8.34E-4
Average cluster diameter in RT	0.0778	0.1060
Computing time [s]	2342	198

General characteristics of the two clustering algorithms on the MitoMix dataset. For computing time details, see Section "Implementation".

The dataset contains 26,051 sequenced peaks arising from 2,589 unique peptides. The peak matching process is performed without knowledge of any peptide identity assignment to a peak. At the conclusion of peak matching, each peptide should be contained in as few clusters as possible--ideally in a single cluster if the tolerances allow it (there are many cases where a peptide either elutes over a long period of time, or elutes at multiple distinct RTs thereby violating the RT tolerance constraint). Table 2 shows the number of known peptides contained in *k *clusters, for *k *= 1, . . . , 9. Clearly, more peptides are contained in a single cluster with MEDEA than with MCLUST.

**Table 2:** MCLUST vs. MEDEA Comparison for MitoMix Data

Number of clusters	MCLUST	MEDEA
1	1788	1955
2	667	571
3	83	42
4	27	13
5	13	3
6	6	5
7	4	0
8	0	0
9	1	0

Mean	1.40	1.28

Number of known peptides contained in *k *clusters, for *k *= 1,. . . , 9, in the MitoMix dataset.

#### The DarTB data set

The DarTB dataset contains a grand total of 653,741 peaks. Again, *m*/*z*, RT and *z *are given for each peak. The half width of the tolerance box was set to *δ*_1 _= 5.96E-6 · *m*/*z *in *m*/*z *(5.96 ppm) and *δ*_2 _= 2.35 in RT, based on actual variation observed for landmark peptides. The general characteristics of the two clustering algorithms are summarized in Table 3.

**Table 3:** MCLUST vs. MEDEA Comparison for DarTB Data

	MCLUST	MEDEA
Number of clusters	287838	218098
Average cluster size	2.27	3.00
Average cluster diameter in *m*/*z*	9.32E-4	0.0023
Average cluster diameter in RT	0.39	0.96
Computing time [s]	663756	17532

General characteristics of the two clustering algorithms on the DarTB dataset. For computing time details, see Section "Implementation".

**Figure 7:**
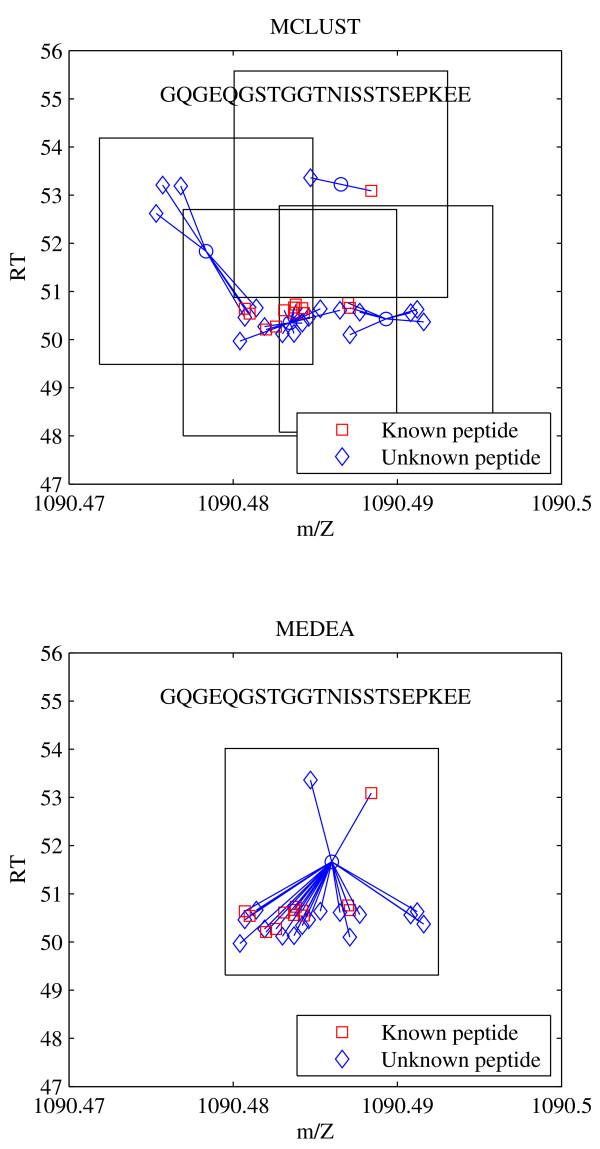
**MCLUST vs. MEDEA Comparison**. Clusters containing all peaks with known peptide GQGEQGSTGGTNISSTSEPKEE. Top: MCLUST, bottom: MEDEA.

In the DarTB data set 39,827 peaks were sequenced from a total of 1,720 unique peptides. Again, peak matching is performed without knowledge of peptide identity, and Table 4 shows the number of known peptides contained in *k *clusters, for *k *= 1, . . . , 10 and *k >*10. 1,162 peptides have the same number of clusters with MCLUST and MEDEA, 547 have more clusters with MCLUST, and only 11 have more clusters with MEDEA. Again, more peptides are contained in a single cluster with MEDEA than with MCLUST. A comparison of the clustering of the peptide GQGEQGSTGGTNISSTSEPKEE is shown in Figure 7. Based on considerations similar to the MitoMix dataset, MEDEA is clearly superior to MCLUST in its ability to cluster (sequenced) peptide landmarks. Since landmarks are a random subset of all peaks in the dataset, the improvement in clustering landmarks afforded by MEDEA should extend to all peaks--identified or otherwise.

**Table 4:** MCLUST vs. MEDEA Comparison for DarTB Data

Number of clusters	MCLUST	MEDEA
1	812	1046
2	489	460
3	215	127
4	98	40
5	46	22
6	24	7
7	10	5
8	7	3
9	3	4
10	7	4
*>*10	9	2

Mean	2.06	1.64

Number of known peptides contained in *k *clusters, for *k *= 1, . . . , 10 and *k >*10, in the DarTB dataset.

## Discussion

### Validation of clustering results

**Figure 8:**
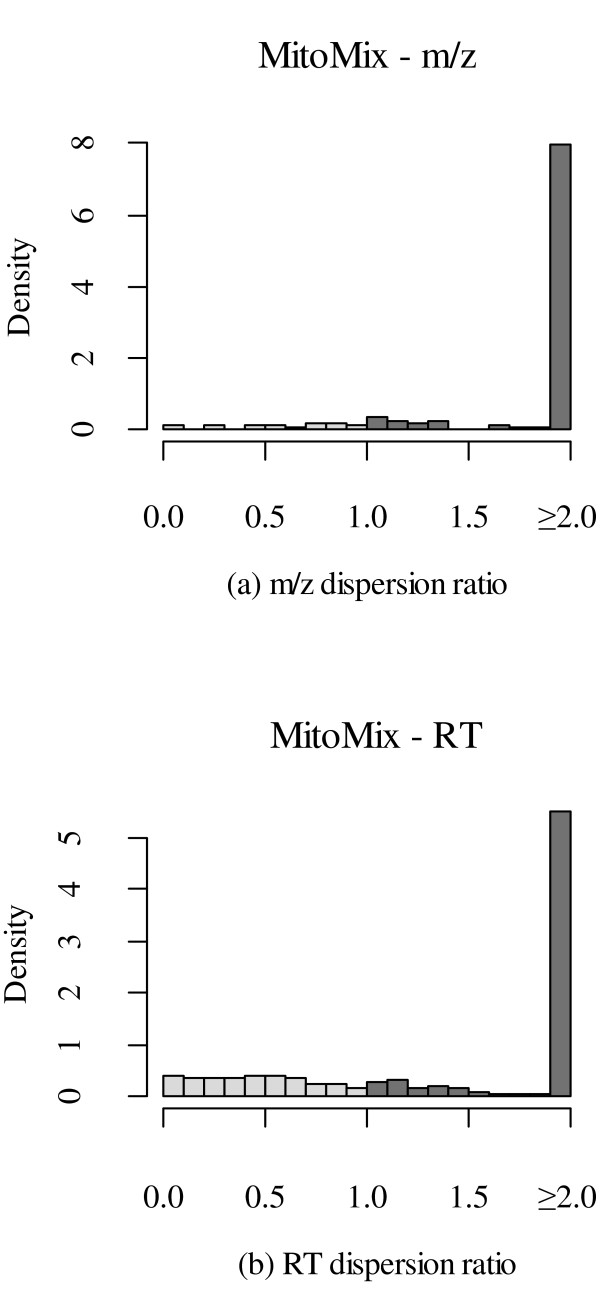
**MCLUST vs. MEDEA Comparison for MitoMix Data**. Histogram of dispersion ratios of (a) *m*/*z *and (b) RT of known peptides due to clustering by MCLUST and MEDEA in MitoMix data. The light/dark gray bins represent respectively lower/higher dispersion of peptides by MCLUST compared to MEDEA. For plotting purposes, ratios greater than 2 are set to 2.

**Figure 9:**
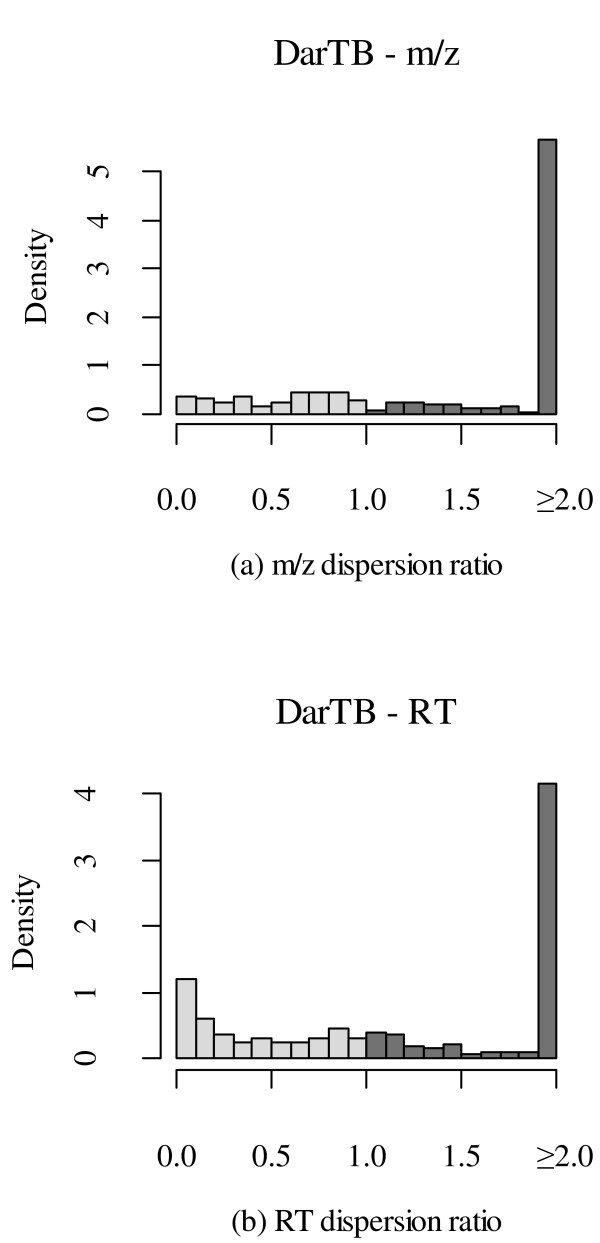
**MCLUST vs. MEDEA Comparison for DarTB Data**. Histogram of dispersion ratios of (a) *m*/*z *and (b) RT of known peptides due to clustering by MCLUST and MEDEA in DarTB data. The light/dark gray bins represent respectively lower/higher dispersion of peptides by MCLUST compared to MEDEA. For plotting purposes, ratios greater than 2 are set to 2.

To determine the quality of our clustering results, we compared the MEDEA and MCLUST clusters of peaks for all known peptides (landmarks) in both datasets. For each known peptide P in a given dataset, we identified all the peaks ∏P of P. We determined the cluster Cπ,A that each peak π ∈ ∏P was assigned to by a particular algorithm A. Then we used the mean 1471-2105-12-358-i6http://www.w3.org/1998/Math/MathMLμCπMathClass-punc,A of the cluster Cπ,A to represent the location locA(π) of each peak π ∈ ∏P , i.e. 1471-2105-12-358-i7http://www.w3.org/1998/Math/MathMLtexttextsfsans-seriflotexttextsfsans-serifcAMathClass-open(πMathClass-close)MathClass-rel=μCπMathClass-punc,A, for both dimensions m/z and RT. Finally, we computed the standard deviation σP,A of all peak-locations locA(∏P ) = {locA(π)|π ∈ ∏P } for a particular peptide P as a measure of per peptide dispersion due to the selected algorithm A. Ideally, if all the peaks ∏P of a peptide P correctly cluster together, then the dispersion σP,A should be 0. We computed the dispersion ratio σP,A/σP,A' for every known peptide P using both algorithms A = MCLUST and A' = MEDEA. After removing ties where both *σ_P_*_,_*_A _*and *σ_P_*_,_*_A_*_' _are equal to 0, or when the ratio is equal to 1 ± *ε *(*ε *= 0.05), we plotted the histograms of ratios for *m*/*z *and RT in both datasets (Figures 8 and 9).

Clearly the MEDEA clusters show significantly less dispersion per peptide than MCLUST as observed by the much higher density of ratios greater than 1 (i.e. for the darker bins in the right half of the histograms) in both datasets.

### Implementation

Compared to the MCLUST implementation of peak matching in PEPPeR, MEDEA is significantly more efficient and results in speedups ranging from about 10 for small datasets to about a factor of 40 for large datasets. In order to compare peak matching speed with MCLUST and MEDEA, we time the entire peak matching process which not only includes the actual clustering part, but involves pre- and post-processing steps, many of which require reading and writing large files, in addition to operations on large tables. MCLUST-based clustering cannot be performed in PEPPeR without the use of some of these processing steps, and in order to perform a fair comparison (from the perspective of efficient PEPPeR implementation), we target the entire peak matching process.

For the MitoMix data MCLUST based peak matching was run on a cluster using 50 processes. The total computing time was calculated as the sum of the time taken by each of the processes, and amounted to 2,343 sec. MEDEA based peak matching took 198 sec on one of the cluster nodes, resulting a speedup of 11.8 (Table 1). For the much larger DarTB data set, MCLUST based peak matching, run on 2,500 processors, had a total computing time of 663,756 sec. MEDEA resulted in a speed up of 37.8 with a single node computing time of 17,532 sec (Table 3). With such speedups on large data sets, MEDEA makes it feasible to process and analyze significantly larger data sets.

For these data sets, the PEPPeR pre- and post-processing adds significant amount of computing time, especially because of large file input/output operations. When MEDEA is run without this overhead, the clustering is even more efficient, with approximate computing times of 45 sec and 1,500 sec on a typical desktop computer for the MitoMix and DarTB datasets, respectively.

## Conclusions

Clustering analysis is used for identifying groups of similar points in data in an unsupervised manner. Popular clustering approaches include hierarchical or partitional algorithms such as agglomerative and k-means clustering. In addition, finite mixture models have been used extensively in biology and medicine [12,14,26]. Often semi-supervised approaches are used for imposing various types of constraints on clustering [27]. Yet few algorithms can address the challenge of enforcing constraints on the variance of the clusters, especially in an unsupervised manner. The challenge is clearly not addressed with a sliding window approach that cannot identify the group structure inherent in the data. In particular, the problem assumes further importance--in various fields ranging from engineering to economics and biotechnology--if the required constraints on the variance of a cluster are dependent on its position. For example, for ChIP-Seq experiments, the appearance of tags along the genome could be modeled by a discrete Poisson distribution, thus requiring the variance of a peak to be equal to its mean [28,29].

Here we have presented a new approach based on robust statistics for identifying clusters in continuous data that respect position-specific constraints on cluster-variance. In this regard, we find the application of M-estimators most suitable. In particular, we developed MEDEA as an effective and fast solution to the problem of peak matching in label-free LC-MS data. By analyzing real-life samples, we have shown that MEDEA is not only significantly more efficient (achieving speedups of up to about 40), but also produces clusters that are more coherent. Data points that are known to arise from the same peptide are more consistently grouped into the same cluster when compared to peak matching based on Gaussian mixture model based clustering using MCLUST, as validated by our comparative analysis. Given its potential usefulness for practical proteomic analysis, the MEDEA algorithm has been integrated into the PEPPeR pipeline.

In the context of applying constrained clustering to peak matching in LC-MS data, the choice of constraints is critical. Here, the *m*/*z *and RT variation tolerance values provide the constraints that MEDEA enforces. If the constraints are too wide, then two different peptides could end up being clustered into a single group. On the other hand, constraints that are too small can cause a peptide to be split into two different clusters, thereby making the analysis of such data for purposes like biomarker discovery much more complex and unreliable. In the PEPPeR platform, the *m*/*z *and RT tolerances are determined based on the observed landmark peptides, and set using the *m*/*z *and RT variation ranges for the landmark peptides, after removing outliers. In spite of that, there could be situations where an aberrant peptide elutes over a long period of time, or when multiple isobaric peptides elute within the given RT tolerance window. Under these situations, it would be impossible for any clustering algorithm to correctly group the peptides without knowledge of the actual peptide sequence obtained by tandem MS or other means.

While MEDEA enforces the specified constraints on the variation within a cluster, it does so without assuming an explicit model, Gaussian or otherwise, for the distribution of peaks in the cluster. Clusters members are thus identified solely by the proximity of their peaks, independent of any assumed parametric distribution, as long as they fit into a box of the prescribed size (i.e., satisfy required constraints) around the center of gravity of the cluster. Outliers that respect the constraints are integrated into the cluster with systematic post-processing. While the issue of robustness for cluster outliers, often due to asymmetric or heavy tailed effects, has recently been addressed with new parametric algorithms (e.g., finite mixtures of multivariate skew *t *distributions [30-32]), such robustness would tend to include--rather than exclude--cluster outliers in the heavy-tailed distributions. Furthermore, as in the case of MCLUST, such model-based clustering methods are not capable of enforcing user-specified constraints on cluster extension. Hence, the constraints would again have to be imposed a-posteriori, requiring a computationally expensive split-and-merge algorithm, similar to the one outlined in Figure 1. Therefore we believe that MEDEA, with its unique combination of a robust estimator with automatic constraint enforcement, presents a useful and effective approach that fills an important gap in clustering applications.

## List of abbreviations used

ChIP-Seq: chromatin immunoprecipitation sequencing; FTMS: Fourier transform mass spectrometry; LC: liquid chromatography; LC-MS: liquid chromatography based mass spectrometry; LC-MS/MS: liquid chromatography based tandem mass spectrometry; LTQ: linear trap quadrupole; MCLUST: model based clustering; MEDEA: M-estimator with deterministic annealing; *m*/*z*: mass-to-charge ratio; PEPPeR: platform for experimental proteomic pattern recognition; RT: retention time; *z*: charge

## Authors' contributions

RF developed and implemented the MEDEA algorithm, applied it to the datasets, analyzed the results, and drafted part of the mansucript. DRM incorporated MEDEA into PEPPeR and applied it to proteomic data analysis, created the datasets used, and contributed to writing the manuscript. SP conceived the project, conducted validation of clustering results, and wrote part of the manuscript. All authors read and approved the final manuscript.
